# Functional Genome Annotation of *Lactiplantibacillus pentosus* KMU32 Reveals Its Dual Potential as a Starter Culture and Probiotic from Traditional Korean Kimchi

**DOI:** 10.4014/jmb.2603.03032

**Published:** 2026-05-11

**Authors:** Seungyeon Han, Sojeong Heo, Seoung-Eun Oh, Mi-Sun Kwak, Moon-Hee Sung, Do-Won Jeong

**Affiliations:** 1 Department of Food and Nutrition, Dongduk Women’s University, Seoul 02748, Republic of Korea; 2 R&BD Center, Korea Yakult Co., Ltd., Yongin 17086, Republic of Korea; 3 KookminBio Corporation, Seoul 02826, Republic of Korea

**Keywords:** *Lactiplantibacillus pentosus* KMU32, Functional property, Antibacterial activity, Probiotic, Genome, Starter

## Abstract

This study evaluated the potential of *Lactiplantibacillus pentosus* KMU32, isolated from kimchi, as both a starter culture for fermented foods and a probiotic candidate by assessing its safety, enzymatic activity, health-promoting properties, and antimicrobial efficacy. Strain KMU32 exhibited susceptibility to seven antibiotics and showed neither hemolytic activity nor biofilm formation, indicating a favorable safety profile. Compared with the reference strain *L. pentosus* KACC 12428^T^, KMU32 demonstrated significantly enhanced growth and protease activity under saline conditions, maintaining robust growth and protease activity in media supplemented with 3% and 6% NaCl. KMU32 also exhibited stronger antibacterial activity than the reference strain against several foodborne pathogens, including *Bacillus cereus*, *Enterococcus faecalis*, *Alcaligenes xylosoxidans*, *Escherichia coli*, and *Salmonella enterica*. In addition, KMU32 showed improved probiotic-associated functional properties, including higher acid tolerance (94.38% survival at pH 2.5), bile salt tolerance (94.29% survival in 0.3% oxgall), and intestinal adhesion ability (98.47% mucin adhesion rate) compared with the reference strain. Whole-genome sequencing revealed that KMU32 possesses a 3,757,123-bp circular chromosome and three plasmids, with a G+C content of 46.2%. Genome analysis identified genetic determinants supporting its phenotypic traits, including three osmoprotectant uptake systems (OpuA, OpuB, and OpuD), four exopolysaccharide biosynthesis operons, and genes annotated as a putative bacitracin synthase and bacitracin export-related protein. Collectively, these phenotypic and genomic characteristics indicate that *L. pentosus* KMU32 is a safe and functionally robust strain with dual potential as a starter culture for high-salt fermented foods and as a probiotic candidate, supporting its promising application in the food industry.

## Introduction

Kimchi is a collective term for fermented foods made from various vegetables in Korea. The most representative type is baechu-kimchi, which is primarily prepared using kimchi cabbage (*Brassica rapa*) as the main ingredient, along with minor ingredients such as fermented seafood (jeotgal), carrot, garlic chives, and onion. In the case of baechu-kimchi produced without the use of starter cultures, the ingredients and manufacturing methods vary depending on the individual preparing it. Nevertheless, lactic acid bacteria consistently dominate the microbial community of baechu-kimchi. The dominant lactic acid bacteria belong to the genera *Leuconostoc*, *Lactobacillus*, *Weissella*, *Lactococcus*, and *Pediococcus* [1-6] . Among these, the genus Leuconostoc, particularly *Leuconostoc mesenteroides*, predominates during the optimal ripening stage when kimchi exhibits its best sensory quality, and thus has been extensively studied for use as a starter culture in fermented foods [7].

An excellent starter culture for fermented foods is required to enhance the sensory properties of the product, shorten the fermentation period, and inhibit the growth of spoilage and pathogenic microorganisms [8]. In contrast, superior probiotic strains aim to improve health benefits in humans or animals [9]. Therefore, selection of starter cultures involves evaluating enzyme activities, such as protease and lipase, that contribute to sensory quality, as well as antimicrobial activity [10]. For probiotic candidates, resistance to acid and bile and the ability to adhere to the intestinal epithelium are assessed to ensure survival and colonization in the gastrointestinal tract [11]. Although starter cultures and probiotics differ in their primary objectives, food production versus human health—both favor antimicrobial activity, as starter cultures inhibit spoilage and pathogenic microorganisms in fermented foods, while probiotics suppress pathogenic bacteria to establish themselves in the intestine. Thus, antimicrobial activity is a common criterion for both applications. Consequently, lactic acid bacteria that possess dual functionality as both starter cultures and probiotics are highly valuable, as they can be utilized in fermented food production while also providing health-promoting benefits.

Recently, during an investigation of the microbial communities present in commercial and homemade baechu-kimchi produced without starter cultures, we isolated 1,219 lactic acid bacterial strains [3-6] . Among these, we identified *L. pentosus* KMU32, a strain exhibiting dual functionality as both a starter culture and a probiotic. In this study, we compared its activities with those of a reference strain to evaluate its enzymatic properties as a starter culture and its probiotic potential. Furthermore, we aimed to elucidate the genetic basis underlying these functional characteristics through whole-genome analysis.

## Materials and Methods

### Bacterial Strains and Culture Conditions

Strain KMU32, originally isolated from baechu-kimchi, was subjected to phenotypical trait and genomic analysis. And to determine whether the observed traits are strain-specific or species-specific, the type strain *L. pentosus* KACC 12428^T^ was used for comparative analysis. This strain was considered an appropriate control because its genome has already been reported (GenBank accession numbers CP032757 and CP032758), facilitating genomic comparisons. *L. pentosus* were cultured in de Man–Rogosa–Sharpe broth (MRS; MB Cell, Republic of Korea) at 30°C for 24 h.

### Antibiotic Susceptibility Test

Antibiotic susceptibility was determined using the Minimum Inhibitory Concentration (MIC) tests. The MICs were determined using the broth microdilution method for seven antibiotics (ampicillin, chloramphenicol, clindamycin, erythromycin, gentamicin, kanamycin, and tetracycline) [12]. Antibiotics were prepared in serial twofold working dilutions in deionized water, with final concentrations in each 96-microwell plate ranging from 0.5 to 64 mg/L. Bacterial strains were cultured twice in MRS broth and adjusted to a 0.5 McFarland turbidity standard. Subsequently, each suspension was diluted 1:100 in Brain Heart Infusion broth (BHI; Becton, Dickinson and Co., USA) to attain the proper inoculum concentration. The final inoculum density reached 5 × 10^5^ colony-forming units/ml, and 200 μL was transferred to each well of the 96-microwell plate and incubated at 30°C for 24 h. The MIC results were verified through at least three independently performed tests. All experiments were conducted at least three times on separate days. Strains with the MICs exceeding the breakpoint are considered resistant [13] .

### Hemolytic and Biofilm-Forming Abilities

Hemolytic activity was evaluated using tryptic soy agar (TSA; Becton, Dickinson and Co.) supplemented with 5% (v/v) rabbit blood (MB Cell) for α-hemolysis and 5% (v/v) sheep blood (MB Cell) for β-hemolysis. For α-hemolytic activity, plates were incubated at 37°C for 24 h. β-Hemolytic activity was assessed by subjecting the plates to a cold shock at 4°C for an additional 24 h following incubation at 37°C for 24 h, as previously described [14]. Hemolytic activity was determined by the presence of clear lytic zones surrounding bacterial colonies on blood agar plates. *Staphylococcus aureus* USA300-p23 and RN4220 were used as positive and negative controls, respectively, for hemolysis assays [15]. All experiments were performed at least three times on separate days.

Biofilm formation by *L. pentosus* strains was assessed using a crystal violet staining method. Briefly, strains were pre-cultured in MRS broth and then inoculated into in tryptic soy broth (TSB; Becton, Dickinson and Co.) supplemented with 1% (w/v) sucrose in 96-well polystyrene microplates. The cultures were incubated at 37°C for 24 h under static conditions. After incubation, wells were gently washed twice with phosphate-buffered saline (PBS, pH 7.4) to remove planktonic cells and air-dried. Biofilms were stained with 0.1% (w/v) crystal violet solution for 3 h at room temperature. Excess stain was removed, and the bound crystal violet was solubilized by adding 300 μL of 95% (v/v) ethanol. Absorbance was measured at 570 nm (OD_570_) using a microplate reader. *S. aureus* USA300-p23 and RN4220 were used as positive and negative controls, respectively. The biofilm assay was conducted in three independent experiments, with each analysis performed in triplicate to assess intra-assay variability.

### Determination of Salt Tolerance and Enzymatic Activities

Salt tolerance of the *L. pentosus* strain was evaluated by monitoring growth kinetics in MRS broth supplemented with NaCl at final concentrations of 0%, 3%, and 6% (w/v). Cultures were incubated at 37°C, and bacterial growth was monitored at regular time intervals by measuring the optical density at OD_600_ using a Synergy HTX microplate reader (BioTek, USA).

Protease activity was assessed on TSA supplemented with 2% (w/v) skim milk and 0.5% (w/v) glucose. Lipase activity was evaluated using tributyrin agar (Sigma-Aldrich, USA) containing 1% (v/v) tributyrin. The tributyrin-supplemented medium was emulsified by sonication prior to autoclaving to ensure uniform dispersion. For enzymatic activity assays, colonies grown on TSA were transferred onto each substrate-supplemented agar plate and incubated at 37°C for 24 h. Enzymatic activity was determined based on the relative diameter of the clear zone formed around each colony. Amylase activity was determined using TSA supplemented with 1% (w/v) soluble starch. After incubation at 37°C for 24 h, the plates were flooded with 1% (w/v) iodine solution, and amylase activity was confirmed by the formation of clear zones surrounding the colonies.

### Antibacterial Activity

The antimicrobial activity of *L. pentosus* KMU32 was evaluated against nine spoilage or foodborne pathogenic bacteria using the agar well diffusion method. The indicator strains used in this study were *Bacillus cereus* KCCM 11341, *Enterococcus faecalis* KCTC 2011, *Listeria monocytogenes* ATCC 19111, *Staphylococcus aureus* ATCC 12692, *Alcaligenes xylosoxidans* KCCM 40240, *Escherichia coli* O157:H7 EDL 933, *Flavobacterium* sp. KCCM 11374, *Salmonella enterica* KCCM 11862, and *Vibrio parahaemolyticus* KCTC 2729. The indicator strains were cultivated overnight in TSB and subsequently inoculated (1%, v/v) into fresh TSB medium. Cultures were incubated until they reached an optical density at OD_600_ of 1.0. Thereafter, 200 μL of each indicator strain culture was evenly spread onto TSA plates. Wells with a diameter of 6 mm were aseptically punched into the agar using a sterile cork borer, and 50 μL of concentrated cell-free supernatant was added to each well. The cell-free supernatant of *L. pentosus* KMU32 was obtained by culturing the strain in MRS broth at 30°C for 24 h, followed by centrifugation to remove bacterial cells. The resulting supernatant was concentrated fourfold using a centrifugal vacuum concentrator (HyperVAC VC2124, Hanil Scientific Inc., Republic of Korea). The inoculated plates were incubated at 30°C for 24 h, and antimicrobial activity was evaluated by measuring the relative diameter of the clear zone formed around each well. All experiments were conducted in triplicate and performed independently.

### Acid Tolerance, Bile Tolerance, and Intestinal Adhesion Abilities

Acid tolerance was evaluated by first culturing *L. pentosus* strains in MRS broth until they reached an optical density at OD_600_ of approximately 0.7. The cultures were then inoculated (1%, v/v) into MRS broth adjusted to pH 2.5 using HCl and incubated at 30°C for 3 h. Viable cell counts were determined and expressed as colony-forming units per milliliter (CFU/mL). As a control, the same procedure was performed using non-adjusted MRS broth (pH 6.5). Acid tolerance was evaluated by comparing the viable cell counts obtained from the acidified medium with those from the control medium.

Bile tolerance was assessed using a similar procedure. Briefly, cultures grown in MRS broth to an OD_600_ of approximately 0.7 were inoculated (1%, v/v) into MRS broth supplemented with 0.3% (w/v) oxgall (MB Cell) and incubated at 30°C for 6 h. Viable cell counts (CFU/mL) were determined after incubation. Control cultures were grown under identical conditions in MRS broth without oxgall. Bile tolerance was evaluated based on the survival of the strains in oxgall-supplemented medium relative to the control medium.

Intestinal adhesion ability was evaluated using a mucin-binding assay, modified from the method described by [16] . Briefly, 100 μL of porcine stomach mucin (Sigma-Aldrich) at a concentration of 10 mg/mL was added to each well of a 96-well microplate and incubated at 4°C for 24 h. After incubation, the wells were washed twice with 0.85% (w/v) NaCl solution. Bacterial cultures grown in MRS broth to an OD_600_ of approximately 0.7 were added (100 μL) to the mucin-coated wells and incubated at 30°C for 2 h. Non-adherent cells were removed by centrifugation at 1,500 × g for 2 min, and the supernatant was discarded. The remaining pellets were washed five times with 0.85% (w/v) NaCl to remove loosely attached cells. Subsequently, 0.1% (w/v) Triton X-100 (Sigma-Aldrich) was added to each well to detach the adherent cells. The recovered cells were serially diluted tenfold in 0.85% NaCl, spread onto MRS agar plates, and incubated at 30°C for 48 h for colony enumeration. Intestinal adhesion ability was expressed as the adhesion ratio, defined as the percentage of viable cells recovered from the mucin-coated wells relative to the viable cell count obtained from blank wells containing MRS broth without mucin. All experiments were performed in three independent experiments, and each analysis was conducted in triplicate to evaluate intra-assay variability.

### Genome Sequencing and Annotation

Whole-genome sequencing of strain KMU32 was performed by CJ Bioscience, Inc. (Republic of Korea) using the PacBio Sequel platform (Pacific Biosciences, USA) with the Single-Molecule Real-Time (SMRT) sequencing system and a 10-kb library. A total of 731,624 reads were generated, providing approximately 403.75-fold genome coverage. The sequencing reads were assembled into a single circular contig using Unicycler (version 0.4.9). Assembly quality assessment and visualization were conducted using CLC Genomics Workbench version 7.5.1 (CLC Bio, Denmark).

Genome annotation was performed using the NCBI Prokaryotic Genome Annotation Pipeline (PGAP; version 4.6). Open reading frames (ORFs) were predicted using Glimmer 3 and functionally annotated by comparison against the Clusters of Orthologous Groups (COG) database and the SEED subsystem database using the RAST server (accessed on September 1, 2025).

### Comparative Genome Analysis

Genome sequence data of *L. pentosus* strain KACC 12428^T^ were retrieved from the National Center for Biotechnology Information (NCBI) genome database for comparative genomic analysis. Core-genome and pan-genome analyses were performed using the Efficient Database framework for comparative Genome Analyses using BLASTP score Ratios (EDGAR) platform, as described by [17]. Comparative analyses at the protein level were conducted based on an all-against-all BLASTP comparison of the annotated genomes to identify shared and strain-specific genes.

### Statistical Analysis

Statistical analyses were performed using the SPSS software (version 29.0; IBM, USA). Data are presented as mean ± standard deviation (SD). Differences among mean values for biofilm formation were evaluated using one-way analysis of variance (ANOVA), followed by Duncan’s multiple range test. Independent t-test was used to compare the mean values of salt tolerance, protease activity, antibacterial activity, acid tolerance, bile tolerance, and intestinal adhesion ability between groups. Statistical significance was determined at *p* < 0.05, *p* < 0.01, and *p* < 0.001, indicated by *, **, and ***, respectively.

### Nucleotide Sequence Accession Number

The complete genome sequence of *L. pentosus* KMU32 was deposited in the NCBI under accession number CM135828.

## Results

### Safety Properties of *L. pentosus* Strain KMU32

The safety of *L. pentosus* KMU32 was evaluated based on antibiotic susceptibility, hemolytic activity, and biofilm formation. The European Food Safety Authority (EFSA) provides guidelines for assessing antibiotic resistance in *L. pentosus*, with the aim of evaluating the potential transfer of antibiotic resistance genes from bacteria to humans or animals [12]. These guidelines are based on microbiological cut-off values established by the European Committee on Antimicrobial Susceptibility Testing (EUCAST) through large-scale monitoring data. The antibiotic susceptibility of *L. pentosus* KMU32 was evaluated against seven antibiotics, and the MIC values for all tested antibiotics were below the EFSA-recommended cut-off values (Table 1), indicating that KMU32 is susceptible to these antibiotics.

**Fig. 1:**
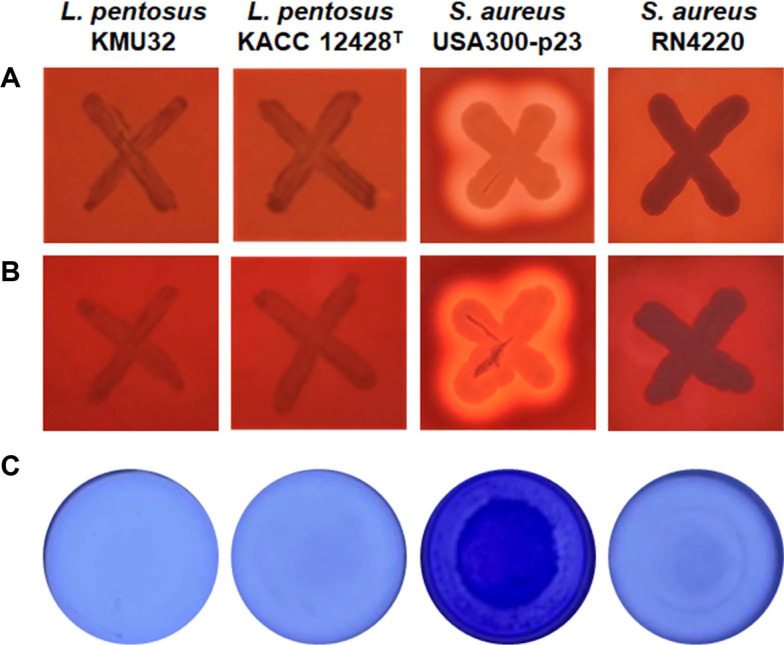
α-Hemolytic activity (A) β-hemolytic activity (B) and biofilm formation (C) of *L. pentosus* KMU32 and KACC 12428^T^. *S. aureus* USA300-p23 and *S. aureus* RN4200 were used as positive and negative controls, respectively. The formation of a clear zone reflects positive hemolysis, and color deposition reflects biofilm formation.

EFSA also maintains a Qualified Presumption of Safety (QPS) list, which includes microbial species considered safe for use in food and feed applications based on criteria such as taxonomic identity, history of safe use, and absence of pathogenicity [18, 19]. *L. pentosus* has been included in the QPS list since its introduction in 2007 [19]. Nevertheless, strain-specific antibiotic resistance must be evaluated prior to industrial application. As *L. pentosus* KMU32 exhibited susceptibility to all tested antibiotics, it can be considered suitable for industrial use. Although no toxic effects have been reported for *L. pentosus* species [20, 21] hemolytic activity was evaluated as part of the standard safety assessment commonly required in Korea. Strain KMU32 exhibited neither α-hemolysis nor β-hemolysis (Fig. 1A and B).

Biofilm formation can enhance virulence when strains possess toxin-related or antibiotic resistance factors. Therefore, the biofilm-forming ability of KMU32 was evaluated. Compared with the positive control strain *Staphylococcus aureus* USA300-p23, *L. pentosus* KMU32 did not form biofilms and showed no statistically significant difference from the negative control (Fig. 1C and S1).

Taken together, these results demonstrate that *L. pentosus* KMU32 is a safe strain, exhibiting susceptibility to seven antibiotics and lacking hemolytic activity and biofilm-forming ability.

### Technological Properties of *L. pentosus* Strain KMU32 as a Starter Culture

To function effectively as a starter culture, strains should possess activities that enhance the sensory properties of fermented foods, such as flavor and aroma. The degradation of macromolecules, including proteins, carbohydrates, and lipids, leads to the production of amino acids, organic acids, and volatile compounds, which contribute to improved sensory quality. Therefore, strains exhibiting enzymatic activities such as protease, amylase, and lipase, as well as acid production, are frequently selected as starter cultures. In addition, because many fermented foods are produced under saline conditions, starter candidates are often required to maintain robust growth and enzymatic activity in the presence of salt. Accordingly, the amylase, protease, and lipase activities, acid production, and salt tolerance of strain KMU32 were evaluated.

**Fig. 2:**
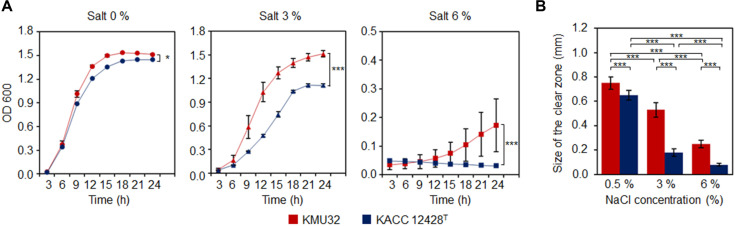
Growth (A) and protease activity (B) of *L. pentosus* KMU32 and KACC 12428^T^ under salt pressure. In (**A**) statistical significance was applied only at 24 h. In (**B**) the formation of a clear zone around the filter paper disc indicates positive enzymatic activity and represents the non-salt-added condition using a medium containing 0.5% salt. Statistical significance was determined at *p* < 0.05, and *p* < 0.001 and indicated asterisks *, and ***.

When growth was monitored under different NaCl concentrations, KMU32 exhibited a higher growth rate than the reference strain KACC 12428^T^ (Fig. 2A). Although the growth rate of KMU32 was reduced to under 3% NaCl compared with salt-free conditions, the final biomass at 24 h did not differ significantly from that observed in the absence of salt. These results indicate that KMU32 exhibits superior growth performance under saline conditions compared with KACC 12428^T^, suggesting its suitability for growth in salt-containing fermented foods.

Strain KMU32 did not exhibit detectable amylase or lipase activity; however, it showed strong protease activity even at 6% NaCl. In contrast, the reference strain KACC 12428^T^ exhibited no protease activity at 6% NaCl and only weak activity at 3% NaCl (Fig. 2B). Notably, KMU32 displayed clear protease activity at 3% NaCl and retained protease activity even at 6% NaCl, indicating its ability to degrade proteins under high-salt conditions.

Taken together, these results demonstrate that KMU32 possesses markedly higher protease activity than the reference strain KACC 12428^T^ and maintains enzymatic activity even at 6% NaCl. Furthermore, its ability to grow and exhibit protease activity under high-salt conditions suggests that KMU32 can survive and function effectively in fermented foods with elevated salt concentrations. Consequently, the ability of KMU32 to maintain robust growth and high protease activity even at 6% NaCl strongly supports its suitability as a starter culture for high-salt fermented foods, where enhanced protein degradation and increased amino acid production are critical determinants of fermentation efficiency and sensory quality.

### Antibacterial Activity of *L. pentosus* Strain KMU32

**Fig. 3:**
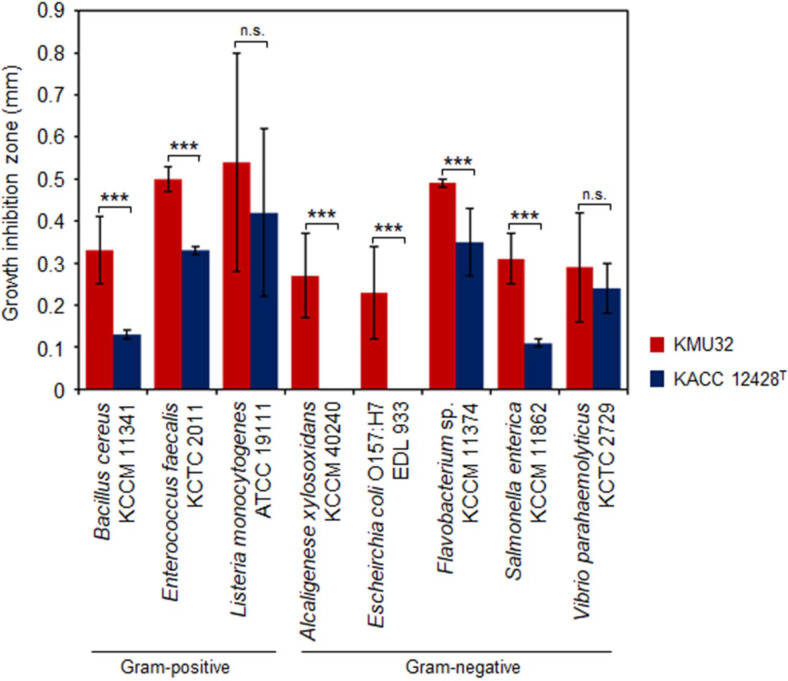
Antibacterial activities of strain KMU32 against food pathogens. Statistical significance was determined at *p* < 0.001 and indicated by an asterisk ***. n.s.: non-significant.

Strains that are safe, exhibit strong enzymatic activity, and possess antibacterial properties fulfill key criteria for application as starter cultures in fermented foods. Accordingly, the antibacterial activity of strain KMU32 was evaluated against nine foodborne pathogenic or spoilage bacteria using the agar well diffusion method (Fig. 3).

Strain KMU32 exhibited growth-inhibitory activity against eight indicator strains tested, with the exception of *S. aureus* ATCC 12692. Notably, compared with KACC 12428^T^, KMU32 demonstrated significantly stronger antibacterial activity against six of the tested foodborne pathogens (*B. cereus* KCCM 11341, *E. faecalis* KCTC 2011, *Flavobacterium* sp. KCCM 11374, *A. xylosoxidans* KCCM 40240, *E. coli* O157:H7 EDL 933, and *V. parahaemolyticus* KCTC 2729). We considered the possibility that the antimicrobial activity was due to the pH of the culture supernatant; however, in our previous study, the pH of the lactic acid bacteria culture supernatant did not exhibit a significant antimicrobial effect [22]. Therefore, our current results further support that the observed antimicrobial activity is not attributable to the pH of the culture supernatant of strain KMU32. Instead, these findings suggest that KMU32 exhibits broad-spectrum antibacterial activity against both spoilage-associated and pathogenic bacteria. This effect is likely mediated by specific antimicrobial compounds, such as bacteriocins or other bioactive metabolites, rather than simple acidification. Taken together, the pronounced antibacterial properties of KMU32 highlight its dual functionality as a promising starter culture for fermented food production and as a probiotic candidate capable of effectively suppressing undesirable microorganisms.

### Functional Activity of *L. pentosus* Strain KMU32

Probiotics are defined as live microorganisms that confer health benefits to the host when administered in adequate amounts [21]. To exert beneficial effects on intestinal health, probiotic strains must survive the harsh gastrointestinal environment, including exposure to low pH (≤3.0) and bile salts, and subsequently adhere to the intestinal epithelium. Therefore, acid tolerance, bile salt tolerance, and intestinal adhesion ability were evaluated as fundamental probiotic properties of strain KMU32.

**Fig. 4:**
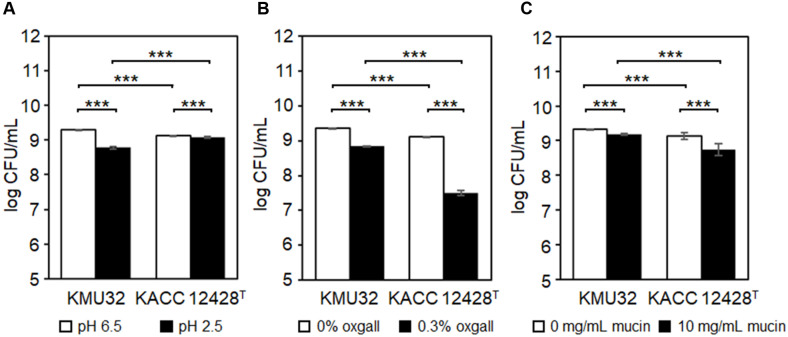
Probiotic potential characterization of *L. pentosus* KMU32. Acid tolerance (A) Bile salt tolerance (B) and Intestinal adhesion ability(C). Statistical significance was determined at *p* < 0.001 and indicated by an asterisk ***.

Acid tolerance was assessed by comparing viable cell counts in MRS broth adjusted to pH 2.5 with those in non-adjusted MRS broth (pH 6.5) [23]. Strain KMU32 exhibited viable counts of 8.78 ± 0.04 log CFU/mL at pH 2.5 and 9.31 ± 0.00 log CFU/mL at pH 6.5, corresponding to a survival rate of 94.38%. In contrast, the reference type strain KACC 12428^T^ showed viable counts of 9.07 ± 0.02 log CFU/mL at pH 2.5 and 9.13 ± 0.01 log CFU/mL at pH 6.5, resulting in a survival rate of 99.42%. These results indicate that KMU32 exhibited slightly lower acid tolerance than the reference strain, although it maintained high survival under acidic conditions (Fig. 4A).

Bile salt tolerance was evaluated using 0.3% (w/v) oxgall [24], and survival was determined by comparison with cultures grown in oxgall-free medium. Strain KMU32 showed viable counts of 8.84 ± 0.01 log CFU/mL in oxgall-supplemented medium and 9.37 ± 0.00 log CFU/mL in the control medium, corresponding to a survival rate of 94.29%. In contrast, KACC 12428^T^ exhibited viable counts of 7.50 ± 0.07 log CFU/mL and 9.11 ± 0.00 log CFU/mL in oxgall-supplemented and control media, respectively, resulting in a survival rate of 82.26%. These results demonstrate that KMU32 possesses significantly higher bile salt tolerance than the reference strain (Fig. 4B).

In addition to surviving gastrointestinal stress, the ability to adhere to the intestinal surface is critical for probiotic persistence and functionality. Intestinal adhesion ability was indirectly evaluated using a mucin-binding assay [16]. The viable cell counts of KMU32 recovered from mucin-coated wells (10 mg/mL) was 9.18 ± 0.02 log CFU/mL, whereas the viable count from non-coated control wells was 9.33 ± 0.00 log CFU/mL, corresponding to an adhesion rate of 98.47%. This adhesion rate was higher than that observed for the reference strain (95.77%) (Fig. 4C).

Taken together, these results demonstrate that strain KMU32 exhibits superior bile salt tolerance and intestinal adhesion ability compared with the reference strain KACC 12428^T^. Although its acid tolerance was slightly lower than that of the reference strain, KMU32 maintained a high survival rate of 94.38% under acidic conditions, satisfying the fundamental requirements for probiotic functionality. These findings suggest that KMU32 represents a promising probiotic candidate, and further investigation of its strain-specific health-promoting effects may support its development as a functionally enhanced probiotic strain.

### General Genome Properties of *L. pentosus* Strain KMU32

To elucidate the genetic basis underlying the safety, enzymatic activity, antibacterial activity, and functional properties of strain KMU32, whole-genome analysis was performed. The complete genome of *L. pentosus* KMU32 consists of a 3,757,123-bp circular chromosome and three plasmids. The genomic G+C content is 46.2%. The genome contains 16 rRNA genes and 71 tRNA genes, and a total of 3,378 open reading frames (ORFs) were predicted.

Functional annotation based on the Clusters of Orthologous Groups (COG) database assigned putative functions to 3,029 genes. Excluding the category “function unknown”, the most abundant functional category was carbohydrate transport and metabolism (342 genes, 11.3%), followed by transcription (260 genes, 8.6%) and amino acid transport and metabolism (232 genes, 7.7%).

Based on SEED subsystem annotation, 1,021 genes were classified into functional subsystems. The most enriched subsystem category was carbohydrates (214 genes, 21.0%), followed by protein metabolism (170 genes, 16.7%). These genomic features are consistent with the observed technological and probiotic properties of KMU32, particularly its carbohydrate utilization capacity and proteolytic activity.

### Genomic Insights into *L. pentosus* Strain KMU32

**Safety Properties of Strain KMU32.** Strain KMU32 exhibited susceptibility to seven antibiotics (ampicillin, chloramphenicol, clindamycin, erythromycin, gentamicin, kanamycin, and tetracycline) and did not show hemolytic activity (Table 1, Fig. 1A and 1B). To genetically, substantiate these safety-related phenotypes, toxin-associated genes and antibiotic resistance genes were analyzed in the genome of strain KMU32.

To identify antibiotic resistance determinants, two bioinformatic tools were employed: ResFinder and the Comprehensive Antibiotic Resistance Database (CARD) algorithms. Analysis using ResFinder (version 4.7.2) predicted the absence of any acquired antibiotic resistance genes in the KMU32 genome (Table S2). In contrast, CARD analysis identified a single gene annotated as *vanY* (ACXXIO_RS04625). The *vanY* gene encodes a zinc-dependent D-Ala-D-Ala carboxypeptidase (EC 3.4.17.14) and is considered an accessory gene associated with vancomycin resistance rather than an essential determinant [25]. Vancomycin resistance typically requires the presence of the *vanHAX* operon, which mediates D-Ala-D-Lac synthesis in peptidoglycan, along with the regulatory genes *vanSR* [25]. Notably, strain KMU32 does not harbor these core vancomycin resistance genes, indicating a negligible likelihood of vancomycin resistance. Taken together, results from both ResFinder and CARD analyses support the absence of functionally relevant antibiotic resistance genes in strain KMU32.

The presence of toxin-related genes was further investigated using VirulenceFinder (Server 2.0), which revealed no virulence genes in the KMU32 genome (Table S3). A keyword-based search using the term “toxin” identified a gene annotated as hemolysin-3 protein (ACXXIO_RS14840). However, our previous studies demonstrated that hemolysin-3 proteins are not directly involved in hemolytic activity [26-28]. Consistent with these findings, strain KMU32 exhibited no hemolysis in phenotypic assays. Moreover, hemolysin-3 homologs have been detected in multiple lactic acid bacterial species without any association with hemolytic phenotypes [26-29] . Therefore, these results collectively confirm that strain KMU32 does not harbor functional toxin genes, including hemolysin-related determinants.

**Technological Properties of Strain KMU32.** Strain KMU32 exhibited protease activity at 3% NaCl and maintained growth even at 6% NaCl, demonstrating superior technological performance under saline conditions compared with the reference type strain KACC 12428^T^. Specifically, KMU32 showed higher protease activity and growth under salt-stressed conditions than the reference strain (Fig. 2). To investigate the genetic basis underlying these technological properties, genome-based analyses were conducted. Analysis of protease-related genes in the KMU32 genome identified a total of 61 putative protease genes, including 55 genes assigned with Enzyme Commission (EC) numbers and six genes without EC annotation (Table S4). Comparative analysis revealed that one protease-related gene (ACXXIO_RS11110) was uniquely present in strain KMU32 and absent in the reference strain. This strain-specific gene may contribute to the enhanced protease activity observed in KMU32 under 3% and 6% NaCl conditions; however, further functional studies are required to confirm its precise role.

**Fig. 5:**
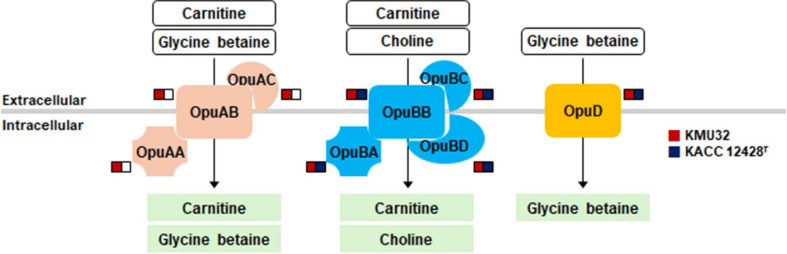
Predicted membrane transport systems and synthesis pathways for osmoprotectants in the *L. pentosus* KMU32 and KACC 12428^T^ genomes. Osmoprotectants are depicted in green. Colored boxes indicate the presence of the corresponding genes, whereas white boxes indicate the absence of the genes. Red and blue represent genes from KMU32 and KACC 12428^T^, respectively.

To further explain the salt tolerance of strain KMU32, genes associated with osmoprotection were examined. Under high-salt conditions, microorganisms commonly accumulate compatible solutes such as choline, glycine betaine, proline betaine, and trehalose to maintain cellular homeostasis [30, 31]. Genome analysis revealed that KMU32 possesses three osmoprotectant uptake systems—OpuA, OpuB, and OpuD—responsible for the transport of compatible solutes into the cell. Comparative genomic analysis showed that the OpuA system was uniquely present in strain KMU32, whereas it was absent in the reference strain (Fig. 5 and Table S5).

Consistent with these genomic findings, strain KMU32 exhibited higher growth rates than KACC 12428^T^ in media containing 3% and 6% NaCl (Fig. 2). These results suggest that the presence of an additional osmoprotectant uptake system, particularly OpuA, may contribute to the enhanced salt tolerance and technological robustness of KMU32 under high-salt fermentation conditions.

**Genes Related to Functional Properties.** Strain KMU32 exhibited superior bile salt tolerance and intestinal adhesion abilities compared with the reference strain KACC 12428^T^ (Fig. 4). To identify genetic determinants potentially contributing to these functional properties, comparative genome analysis was conducted based on genes previously reported to be associated with bile tolerance, acid stress resistance, and intestinal adhesion [32-34].

**Fig. 6:**
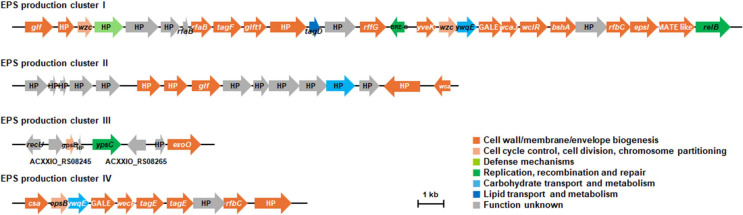
Annotated genes related to the exopolysaccharide production of strain KMU32. The position and orientation of the coding regions are presented by arrows. The name of the arrows is the annotated gene name or the locus number. Each color represents a functional classification based on the COG category. HP, hypothetical protein.

Exopolysaccharides (EPS) form a protective surface layer in bacteria and are known to contribute to survival under harsh environmental conditions as well as adhesion to epithelial surfaces, including the intestinal tract [32, 35]. Genome analysis revealed that strain KMU32 harbors four EPS-related operons involved in exopolysaccharide biosynthesis (Fig. 6; Table S6), suggesting a potential role in enhanced stress tolerance and intestinal adhesion.

In addition to EPS-related genes, KMU32 was examined for the presence of genes previously reported to be associated with survival under acid and bile stress conditions in lactic acid bacteria [33, 34]. Comparative analysis confirmed that KMU32 possesses all stress-related genes reported by [33] (Table S7). Moreover, KMU32 was found to harbor two additional genes do not present in the reference strain: an H^+^/Cl^−^ exchange transporter (*clcA*, ACXXIO_RS16715) and an oligopeptide-binding protein (*oppA*, ACXXIO_RS00970). Although the presence of these additional genes alone cannot fully explain the superior acid and bile tolerance of KMU32 compared with the reference strain, their presence suggests a possible contribution to stress resistance and intestinal persistence. Further functional studies are required to clarify the roles of these genes.

**Antibacterial Property of *L. pentosus* Strain KMU32.** *L. pentosus* strain KMU32 exhibited antagonistic activity against pathogenic and/or spoilage microorganisms associated with food, as determined using the agar well diffusion method. Antibacterial activity was observed against seven foodborne pathogens, including *Bacillus cereus*, *Enterococcus faecalis*, *Flavobacterium* sp., *Listeria monocytogenes*, *S. aureus*, *Salmonella enterica*, and *Vibrio parahaemolyticus* (Fig. 3). To investigate the genetic basis underlying this antibacterial activity, genome-based analysis was performed.

Genome annotation revealed that strain KMU32 harbors a gene annotated as bacitracin synthase (ACXXIO_RS15735) as well as a gene encoding a bacitracin export permease, BceB (ACXXIO_RS12490). According to previous studies, effective bacitracin-mediated antimicrobial activity generally requires not only bacitracin synthase but also a complete bacitracin ATP-binding cassette (ABC) transporter system for secretion, along with a two-component regulatory system (*bacRS*) responsible for controlling gene expression [36, 37] . Accordingly, we examined the genes surrounding the annotated locus and searched for homologous genes in the genome of strain KMU32 using the amino acid sequences of previously reported bacitracin transporter genes. However, strain KMU32 does not appear to possess the full complement of genes constituting this canonical bacitracin biosynthesis and regulatory system. Despite the absence of a complete bacitracin-associated gene cluster, the presence of genes annotated as bacitracin synthase and bacitracin export-related proteins suggest a potential link to the observed antibacterial activity. The functional role of these genes in KMU32, as well as their contribution to antimicrobial activity, remains unclear and warrants further investigation.

## Discussion

In this study, *L. pentosus* KMU32 was systematically evaluated as a potential starter culture and probiotic candidate through phenotypic characterization and genome-based analysis. The results demonstrate that KMU32 possesses a combination of safety, technological robustness, antibacterial activity, and probiotic-associated functional properties, supporting its dual applicability in fermented food production and host health promotion.

Safety assessment revealed that KMU32 was susceptible to all seven antibiotics tested and exhibited neither hemolytic activity nor biofilm formation, fulfilling key safety requirements for food-related applications. Genome analysis further supported these findings by confirming the absence of acquired antibiotic resistance genes and virulence-associated determinants. Although a *vanY*-like gene was detected through CARD analysis, the absence of core vancomycin resistance genes (*vanHAX* and *vanSR*) strongly suggests that KMU32 does not pose a risk of vancomycin resistance. These results collectively indicate that KMU32 is a safe strain suitable for industrial use.

From a technological perspective, KMU32 exhibited superior growth and protease activity under high-salt conditions compared with the reference strain KACC 12428^T^. Notably, KMU32 maintained protease activity even at 6% NaCl, a condition relevant to many fermented foods, including kimchi. Genome analysis revealed a higher number of protease-related genes in KMU32, including one strain-specific protease gene, which may contribute to enhanced protein degradation. In addition, the presence of multiple osmoprotectant uptake systems, particularly the OpuA system uniquely found in KMU32, provides a plausible genetic explanation for its improved salt tolerance. These features suggest that KMU32 is well adapted to saline fermentation environments and may enhance amino acid release and flavor development during fermentation.

KMU32 also exhibited broad-spectrum antibacterial activity against multiple foodborne pathogens and spoilage microorganisms. While genome annotation identified genes annotated as bacitracin synthase and a bacitracin export-related protein, the absence of a complete bacitracin biosynthesis and regulatory gene cluster indicates that the observed antibacterial activity may not be solely attributable to canonical bacitracin production. Instead, organic acid production, synergistic antimicrobial metabolites, or uncharacterized antimicrobial peptides may contribute to this phenotype. Further studies are required to elucidate the precise antibacterial mechanisms employed by KMU32.

Regarding probiotic functionality, KMU32 demonstrated high survival rates under acidic and bile salt conditions, as well as superior intestinal adhesion ability compared with the reference strain. Genome analysis revealed multiple EPS biosynthesis operons and stress-related genes associated with acid and bile tolerance. Additionally, KMU32 harbored strain-specific genes, including *clcA* and *oppA*, which may contribute to enhanced stress resistance and intestinal persistence. Although direct causality cannot be conclusively established, the concordance between phenotypic traits and genomic features supports the probiotic potential of KMU32.

Overall, the integration of phenotypic assays and genomic analyses provides compelling evidence that KMU32 is a robust strain with dual starter–probiotic functionality, making it a promising candidate for high-salt fermented foods with added health benefits.

In conclusion, *L. pentosus* KMU32 was identified as a safe and functionally robust strain exhibiting desirable properties as both a starter culture and a probiotic candidate. KMU32 demonstrated antibiotic susceptibility, absence of hemolytic activity and biofilm formation, strong protease activity under high-salt conditions, broad-spectrum antibacterial activity, and high tolerance to acidic and bile environments, along with strong intestinal adhesion ability.

Genome-based analysis supported these phenotypic characteristics by revealing the absence of functional antibiotic resistance and virulence genes, enrichment of protease- and osmoprotection-related genes, multiple EPS biosynthesis operons, and stress-response genes associated with gastrointestinal survival. Taken together, these results suggest that KMU32 is well suited for application in high-salt fermented foods and has the potential to confer additional probiotic benefits. Future studies focusing on strain-specific health-promoting effects and fermentation performance in food matrices will further support its industrial and functional applications.

## Supplemental Materials

Supplementary data for this paper are available on-line only at http://jmb.or.kr.



## Figures and Tables

**Table 1:** Minimal inhibitory concentrations of *Lactiplantibacillus pentosus* KMU32 and KACC 12428^T^ against seven antibiotics.

Antibiotics	KMU32	KACC 12428^T^	Breakpoint*
Ampicillin	2	4	2
Chloramphenicol	2	2	8
Clindamycin	0.5	0.5	2
Erythromycin	0.5	0.5	1
Gentamicin	0.5	0.5	16
Kanamycin	2	8	64
Tetracycline	4	4	32

*EFSA Breakpoint for *Lactiplantibacillus pentosus*.
