# Supplementary material for: Functional Genome Annotation of Lactiplantibacillus pentosus KMU32 Reveals Its Dual Potential as a Starter Culture and Probiotic from Traditional Korean Kimchi
Source: J Microbiol Biotechnol. 2026 May 11;36:e2603032. doi: 10.4014/jmb.2603.03032 (PMC13181314; doi:10.4014/jmb.2603.03032)
Supplement: Supplementary file 1 [file jmb-36-e2603032-supple.pdf]

J. Microbiol. Biotechnol. 2026. 36: e2603032
https://doi.org/10.4014/jmb.2603.03032

Supplementary Figure and Tables

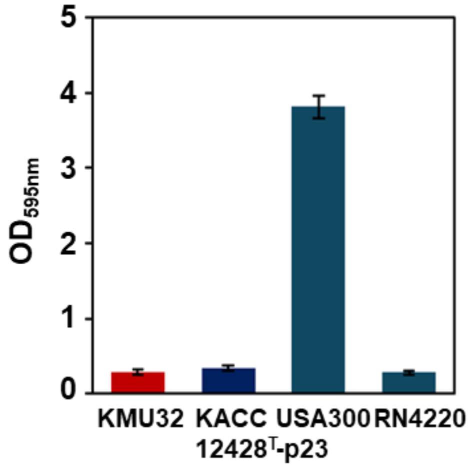

Fig. S1. Quantitative biofilm formation of *L. pentosus* KMU32 and KACC 12428^{T} *S. aureus* USA300-p23 and *S. aureus* RN4200 served as positive and negative controls for hemolytic activities, respectively.

Table S2. List of potential antibiotic resistance activities based on whole-genome sequencing (WGS) using ResFinder.

Class	Antimicrobial	WGS-predicted phenotype
Aminocyclitol	Spectinomycin	No resistance
Aminoglycoside	Amikacin	No resistance
	Apramycin	No resistance
	Arbekacin	No resistance
	Astromicin	No resistance
	Butiromycin	No resistance
	Butirosin	No resistance
	Dibekacin	No resistance
	Fortimicin	No resistance
	Gentamicin	No resistance
	Hygromycin	No resistance
	Isepamicin	No resistance
	Kanamycin	No resistance
	Kasugamycin	No resistance
	Lividomycin	No resistance
	Neomycin	No resistance
	Netilmicin	No resistance
	Paromomycin	No resistance
	Ribostamycin	No resistance
	Sisomicin	No resistance
	Streptomycin	No resistance
	Tobramycin	No resistance
	Unknown aminoglycoside	No resistance
Amphenicol	Chloramphenicol	No resistance
	Florfenicol	No resistance
Beta-lactam	Amoxicillin	No resistance
	Amoxicillin+Clavulanic acid	No resistance
	Ampicillin	No resistance
	Ampicillin+Clavulanic acid	No resistance
	Aztreonam	No resistance
	Cefepime	No resistance
	Cefixime	No resistance
	Cefotaxime	No resistance
	Cefotaxime+Clavulanic acid	No resistance
	Cefoxitin	No resistance
	Ceftazidime	No resistance

Class	Antimicrobial	WGS-predicted phenotype
	Ceftazidime+Avibactam	No resistance
	Ceftriaxone	No resistance
	Cephalothin	No resistance
	Ertapenem	No resistance
	Imipenem	No resistance
	Meropenem	No resistance
	Penicillin	No resistance
	Piperacillin	No resistance
	Piperacillin+Clavulanic acid	No resistance
	Piperacillin+Tazobactam	No resistance
	Temocillin	No resistance
	Ticarcillin	No resistance
	Ticarcillin+Clavulanic acid	No resistance
	Unknown beta-lactam	No resistance
Folate pathway antagonist	Sulfamethoxazole	No resistance
	Trimethoprim	No resistance
Fosfomycin	Fosfomycin	No resistance
Glycopeptide	Bleomycin	No resistance
	Teicoplanin	No resistance
	Vancomycin	No resistance
Ionophores	Maduramicin	No resistance
	Narasin	No resistance
	Salinomycin	No resistance
Lincosamide	Clindamycin	No resistance
	Lincomycin	No resistance
Macrolide	Azithromycin	No resistance
	Carbomycin	No resistance
	Erythromycin	No resistance
	Oleandomycin	No resistance
	Spiramycin	No resistance
	Telithromycin	No resistance
	Tylosin	No resistance
Nitroimidazole	Metronidazole	No resistance
Oxazolidinone	Linezolid	No resistance
Pleuromutilin	Tiamulin	No resistance
Polymyxin	Colistin	No resistance
Pseudomonic acid	Mupirocin	No resistance
Quinolone	Ciprofloxacin	No resistance

Class	Antimicrobial	WGS-predicted phenotype
	Fluoroquinolone	No resistance
	Nalidixic acid	No resistance
	Unknown quinolone	No resistance
Rifampicin	Rifamycin	No resistance
Steroid antibacterial	Fusidic acid	No resistance
Streptogramin A	Dalfopristin	No resistance
	Pristinamyciniia	No resistance
	Quinupristin+Dalfopristin	No resistance
	Virginiamycinm	No resistance
Streptogramin B	Pristinamycinia	No resistance
	Quinupristin	No resistance
	Virginiamycins	No resistance
Tetracycline	Doxycycline	No resistance
	Minocycline	No resistance
	Tetracycline	No resistance
	Tigecycline	No resistance
Under development	Ceftiofur	No resistance

Table S3. List of virulence genes identified using VirulenceFinder.

Gene	Virulence factor of *Lactiplantibacillus pentosus* KMU32
Exoenzyme genes for *Staphylococcus aureus*	No hit found
Hostimm genes for *Staphylococcus aureus*	No hit found
Shiga-toxin genes	No hit found
Toxin genes for *Staphylococcus aureus*	No hit found
Virulence genes for *Enterococcus*	No hit found
Virulence genes for *Enterococcus faecium* & *Enterococcus lactis*	No hit found
Virulence genes for *Escherichia coli*	No hit found
Virulence genes for *Listeria*	No hit found

Table S4. Putative protease genes identified in *L. pentosus* KMU32.

Product	E.C.	Gene locus		Homology (%)
		KMU32	KACC 12428^T	
Dipeptidase A	3.4.-.-	ACXXIO_RS01140	LP314_RS01250	99.8
Glutenin, high molecular weight subunit PW212	3.4.-.-	ACXXIO_RS12785	LP314_RS13210	100
hypothetical protein	3.4.-.-	ACXXIO_RS11100	LP314_RS11500	34.9
Probable dipeptidase	3.4.-.-	ACXXIO_RS01625	LP314_RS01775	100
Probable dipeptidase	3.4.-.-	ACXXIO_RS08025	LP314_RS08290	100
Probable dipeptidase B	3.4.-.-	ACXXIO_RS04395	LP314_RS04585	100
Probable endopeptidase p60	3.4.-.-	ACXXIO_RS05725	LP314_RS05890	99.2
Serine/threonine-rich protein adg2	3.4.-.-	ACXXIO_RS12985	LP314_RS13415	100
Signaling mucin MSB2	3.4.-.-	ACXXIO_RS06170	LP314_RS06380	55.3
Methionyl aminopeptidase	3.4.11.18	ACXXIO_RS01235	LP314_RS01365	100
Membrane alanyl aminopeptidase	3.4.11.2	ACXXIO_RS04350	LP314_RS04535	100
Tripeptide aminopeptidase	3.4.11.4	ACXXIO_RS08850	LP314_RS09155	99.8
Prolyl aminopeptidase	3.4.11.5	ACXXIO_RS02385	LP314_RS02575	99.7
Prolyl aminopeptidase	3.4.11.5	ACXXIO_RS04010	LP314_RS04205	100
Xaa-Pro aminopeptidase	3.4.11.9	ACXXIO_RS07540	LP314_RS07790	100
Dipeptidase E	3.4.13.21	ACXXIO_RS07440	LP314_RS07695	100
D-Ala-D-Ala dipeptidase	3.4.13.22	ACXXIO_RS03630	LP314_RS03810	100
Xaa-Pro dipeptidase	3.4.13.9	ACXXIO_RS10430	LP314_RS10755	100
Xaa-Pro dipeptidase	3.4.13.9	ACXXIO_RS03450	LP314_RS03630	99.9
Xaa-Pro dipeptidyl-peptidase	3.4.14.11	ACXXIO_RS04025	LP314_RS04220	99.7
Serine-type D-Ala-D-Ala carboxypeptidase	3.4.16.4	ACXXIO_RS01295	LP314_RS01430	100
Serine-type D-Ala-D-Ala carboxypeptidase	3.4.16.4	ACXXIO_RS05885	LP314_RS06075	100
Serine-type D-Ala-D-Ala carboxypeptidase	3.4.16.4	ACXXIO_RS06660	LP314_RS06890	99.8
Serine-type D-Ala-D-Ala carboxypeptidase	3.4.16.4	ACXXIO_RS07305	LP314_RS07560	100
Serine-type D-Ala-D-Ala carboxypeptidase	3.4.16.4	ACXXIO_RS13905	LP314_RS14330	100
Serine-type D-Ala-D-Ala carboxypeptidase	3.4.16.4	ACXXIO_RS14385	LP314_RS14810	96.1
Serine-type D-Ala-D-Ala carboxypeptidase	3.4.16.4	ACXXIO_RS05740	LP314_RS05905	98.5
Serine-type D-Ala-D-Ala carboxypeptidase	3.4.16.4	ACXXIO_RS11005	LP314_RS11375	100
Serine-type D-Ala-D-Ala carboxypeptidase	3.4.16.4	ACXXIO_RS14945	LP314_RS15375	100
Muramoyltetrapeptide carboxypeptidase	3.4.17.13	ACXXIO_RS08280	LP314_RS08555	100
Zinc D-Ala-D-Ala carboxypeptidase	3.4.17.14	ACXXIO_RS04625	LP314_RS04820	100
Cell wall integrity and stress response component	3.4.21.-	ACXXIO_RS09805	LP314_RS10120	100
C-terminal processing peptidase	3.4.21.102	ACXXIO_RS08675	LP314_RS08960	100
Rhomboid protease	3.4.21.105	ACXXIO_RS07425	LP314_RS07680	100
Peptidase Do	3.4.21.107	ACXXIO_RS00180	LP314_RS00290	100
Signal peptidase I	3.4.21.89	ACXXIO_RS01545	LP314_RS01695	100

Product	E.C.	Gene locus		Homology (%)
		KMU32	KACC 12428^T	
Signal peptidase I	3.4.21.89	ACXXIO_RS13080	LP314_RS13505	100
Signal peptidase I	3.4.21.89	ACXXIO_RS13085	LP314_RS13510	100
Signal peptidase I	3.4.21.89	ACXXIO_RS16570	LP314_RS16780	100
Endopeptidase Clp	3.4.21.92	ACXXIO_RS02910	LP314_RS03085	100
Endopeptidase Clp	3.4.21.92	ACXXIO_RS03700	LP314_RS03885	100
Endopeptidase Clp	3.4.21.92	ACXXIO_RS06445	LP314_RS06670	98.3
Signal peptidase II	3.4.23.36	ACXXIO_RS08345	LP314_RS08625	100
Type 4 prepilin-like protein leader peptide-processing enzyme	3.4.23.43 , 2.1.1.-	ACXXIO_RS04700	LP314_RS04895	100
ATP-dependent zinc metalloprotease FtsH	3.4.24.-	ACXXIO_RS02450	LP314_RS02645	100
Group B oligopeptidase PepB	3.4.24.-	ACXXIO_RS10145	LP314_RS10485	100
Oligoendopeptidase F like protein	3.4.24.-	ACXXIO_RS12000	LP314_RS12420	100
Phage-like element PBSX protein XkdO	3.4.24.-	ACXXIO_RS11110	-	-
Probable protease eep	3.4.24.-	ACXXIO_RS09400	LP314_RS09710	100
Protease HtpX like protein	3.4.24.-	ACXXIO_RS02285	LP314_RS02475	100
Putative zinc metalloproteinase in scaA 5'region	3.4.24.-	ACXXIO_RS15435	LP314_RS15865	100
Insulysin	3.4.24.56	ACXXIO_RS10640	LP314_RS10990	100
Meprin B	3.4.24.63	ACXXIO_RS13685	LP314_RS14115	100
Membrane-type matrix metalloproteinase-1	3.4.24.80	ACXXIO_RS08950	LP314_RS09250	100
HslU--HslV peptidase	3.4.25.2	ACXXIO_RS08625	LP314_RS08910	100
ATP-dependent protease ATPase subunit HslU		ACXXIO_RS08620	LP314_RS08905	100
Probable inactive metalloprotease YmfF		ACXXIO_RS10645	LP314_RS10995	100
Protein SprT-like		ACXXIO_RS02675	LP314_RS02895	100
Putative membrane peptidase YdiL		ACXXIO_RS03420	LP314_RS03595	100
Putative membrane protease YugP		ACXXIO_RS14705	LP314_RS15135	100
Uncharacterized protein		ACXXIO_RS10320	LP314_RS10650	100

Table S5. Osmoprotectant synthesis- and transporter system-related genes in *L. pentosus* KMU32 genome.

Product	E.C.	COG	Gene	KMU32	KACC 12428^T
Glycine betaine/carnitine transport binding protein GbuC	3.6.3.32	E	*opuAC*	ACXXIO_RS16780	-
Glycine betaine/carnitine transport permease protein GbuB		E	*opuAB*	ACXXIO_RS16775	-
Quaternary-amine-transporting ATPase		E	*opuAA*	ACXXIO_RS16770	-
Methionine import ATP-binding protein MetN		V	*opuBA*	ACXXIO_RS07595	LP314_RS07845
Choline transport system permease protein OpuBB		E	*opuBB*	ACXXIO_RS07600	LP314_RS07850
Carnitine transport binding protein OpuCC		E	*opuBC*	ACXXIO_RS07605	LP314_RS07855
Choline transport system permease protein OpuBD		E	*opuBD*	ACXXIO_RS07610	LP314_RS07860
Glycine betaine transporter OpuD		P	*opuD*	ACXXIO_RS15010	LP314_RS15435

Table S6. Exopolysaccharide (EPS) related genes in *L. pentosus* KMU32 genomes.

Product	E.C.	COG	Gene	KMU32
EPS production cluster I				
UDP-galactopyranose mutase	5.4.99.9	M	*glf*	ACXXIO_RS05400
Hypothetical protein		M		ACXXIO_RS05405
Hypothetical protein	2.7.10.-	D	*wzc*	ACXXIO_RS05410
Hypothetical protein		V		ACXXIO_RS05415
Hypothetical protein		S		ACXXIO_RS05420
Hypothetical protein		S		ACXXIO_RS05425
Hypothetical protein	2.4.1.-		*rfaB*	ACXXIO_RS05430
Lipopolysaccharide 1,6-galactosyltransferase	2.4.1.-	M	*rfaB*	ACXXIO_RS05435
Teichoic acid poly(glycerol phosphate) polymerase	2.7.8.12	M|M	*tagF*	ACXXIO_RS05440
Rhamnopyranosyl-N-acetylglucosaminyl-diphospho-decaprenol beta-1,4/1,5-galactofuranosyltransferase	2.4.1.287	M	*glft1*	ACXXIO_RS05445
Hypothetical protein		M		ACXXIO_RS05450
Glycerol-3-phosphate cytidylyltransferase	2.7.7.39	IM	*tagD*	ACXXIO_RS05455
Hypothetical protein				ACXXIO_RS05460
dTDP-glucose 4,6-dehydratase	4.2.1.46	M	*rffG*	ACXXIO_RS05465
Probable integrase/recombinase YoeC		L		ACXXIO_RS05470
Capsular polysaccharide biosynthesis protein CapA		M		ACXXIO_RS05475
Tyrosine-protein kinase YwqD	2.7.10.-	D	*wzc*	ACXXIO_RS05480
Protein-tyrosine-phosphatase	3.1.3.48	GM		ACXXIO_RS05485
UDP-glucose 4-epimerase	5.1.3.2	M	GALE	ACXXIO_RS05490
Exopolysaccharide production protein PSS		M		ACXXIO_RS05495
UDP-Gal:alpha-D-GlcNAc-diphosphoundecaprenol alpha-1,3-galactosyltransferase	2.4.1.343	M	*wclR*	ACXXIO_RS05500
Hypothetical protein	2.4.1.-	M	*bshA*	ACXXIO_RS05505
Hypothetical protein		S		ACXXIO_RS05510
Uncharacterized protein	2.4.1.-	M	*rfbC*	ACXXIO_RS05515
General stress protein	2.-.-.-	M|M	*epsI*	ACXXIO_RS05520
Hypothetical protein		M		ACXXIO_RS05525
Hypothetical protein		L		ACXXIO_RS05530
EPS production cluster II				
Hypothetical protein				ACXXIO_RS05600
Hypothetical protein				ACXXIO_RS05605
Hypothetical protein				ACXXIO_RS05610
Hypothetical protein				ACXXIO_RS05615
Hypothetical protein				ACXXIO_RS05620
Hypothetical protein		M		ACXXIO_RS05625
Hypothetical protein		M		ACXXIO_RS05630
UDP-galactopyranose mutase	5.4.99.9	M	*glf*	ACXXIO_RS05635
Hypothetical protein				ACXXIO_RS05640
Hypothetical protein				ACXXIO_RS05645

Product	E.C.	COG	Gene	KMU32
Hypothetical protein		S		ACXXIO_RS05650
Hypothetical protein		S		ACXXIO_RS05655
Hypothetical protein		G		ACXXIO_RS05660
Hypothetical protein		S		ACXXIO_RS05665
Hypothetical protein		M		ACXXIO_RS05670
Galactosyl transferase CpsE		M		ACXXIO_RS05675
EPS production cluster III				
Holliday junction resolvase RecU		S		ACXXIO_RS08240
UPF0398 protein		S		ACXXIO_RS08245
Cell cycle protein GpsB		D		ACXXIO_RS08250
Hypothetical protein				ACXXIO_RS08255
Ribosomal RNA large subunit methyltransferase K/L	2.1.1.-	L	*ypsC*	ACXXIO_RS08260
Putative esterase		S		ACXXIO_RS08265
Hypothetical protein		S		ACXXIO_RS08270
Putative glycosyltransferase EpsH	2.4.-.-	M	*exoO*	ACXXIO_RS08275
EPS production cluster IV				
Hypothetical protein		M		ACXXIO_RS09605
Uncharacterized protein	2.4.1.-	M	*rfbC*	ACXXIO_RS09610
Hypothetical protein		S		ACXXIO_RS09615
Poly(glycerol-phosphate) alpha-glucosyltransferase	2.4.1.52	M	*tagE*	ACXXIO_RS09620
Poly(glycerol-phosphate) alpha-glucosyltransferase	2.4.1.52	M	*tagE*	ACXXIO_RS09625
UDP-N-acetylgalactosamine-undecaprenyl-phosphateN-acetylgalactosaminephosphotransferase		M	*wecP*	ACXXIO_RS09630
UDP-glucose 4-epimerase		M	GALE	ACXXIO_RS09635
Protein-tyrosine-phosphatase	3.1.3.48	GM		ACXXIO_RS09640
Non-specific protein-tyrosine kinase	2.7.10.2	D	*epsB*	ACXXIO_RS09645
Capsular polysaccharide biosynthesis protein CapA		M		ACXXIO_RS09650

Table S7. Annotated probiotic properties genes identified in the *L. pentosus* KMU32 genomes.

Product	E.C.	COG	Gene	KMU32	KACC 12428^{T}
Acid stress/Bile resistance					
Phosphoglycerate mutase (2,3-diphosphoglycerate-dependent)	5.4.2.11	G	*gpmA*	ACXXIO_RS03110	LP314_RS03290
Phosphoglycerate kinase	2.7.2.3	G	*pgk*	ACXXIO_RS03735	LP314_RS03920
Phosphopyruvate hydratase	4.2.1.11	G	*eno*	ACXXIO_RS03745	LP314_RS03930
Phosphoglycerate mutase (2,3-diphosphoglycerate-dependent)	5.4.2.11	G	*gpmA*	ACXXIO_RS08375	LP314_RS08655
Chaperone protein DnaK		O		ACXXIO_RS09300	LP314_RS09605
Phosphoglycerate mutase (2,3-diphosphoglycerate-dependent)	5.4.2.11	G	*gpmA*	ACXXIO_RS13110	LP314_RS13535
Phosphoglycerate mutase (2,3-diphosphoglycerate-dependent)	5.4.2.11	G	*gpmA*	ACXXIO_RS14290	LP314_RS14715
BSH					
Choloylglycine hydrolase	3.5.1.24	K		ACXXIO_RS05995	LP314_RS06185
Choloylglycine hydrolase	3.5.1.24	M		ACXXIO_RS11820	LP314_RS12240
Choloylglycine hydrolase	3.5.1.24	S		ACXXIO_RS12275	LP314_RS12695
Choloylglycine hydrolase	3.5.1.24	M		ACXXIO_RS15140	LP314_RS15565
Acid stress					
Lichenan permease IIC component		G		ACXXIO_RS01365	LP314_RS01500
GTP diphosphokinase	2.7.6.5	S		ACXXIO_RS01400	LP314_RS01540
L-lactate dehydrogenase	1.1.1.27	C	*ldh*	ACXXIO_RS01715	LP314_RS01875
L-lactate dehydrogenase	1.1.1.27	C	*ldh*	ACXXIO_RS02405	LP314_RS02595
60 kDa chaperonin		O		ACXXIO_RS03430	LP314_RS03610
Glyceraldehyde-3-phosphate dehydrogenase (phosphorylating)	1.2.1.12	G	*gapA*	ACXXIO_RS03730	LP314_RS03915
H(+)/Cl(-) exchange transporter ClcA		P		ACXXIO_RS03755	LP314_RS03940
L-lactate dehydrogenase	1.1.1.27	C	*ldh*	ACXXIO_RS05045	LP314_RS05240
L-lactate dehydrogenase	1.1.1.27	C	*ldh*	ACXXIO_RS05750	LP314_RS05910
Pyruvate kinase	2.7.1.40	G	*pyk*	ACXXIO_RS08815	LP314_RS09120
Chaperone protein ClpB		O		ACXXIO_RS08845	LP314_RS09150
Undecaprenol kinase	2.7.1.66	M	*dgkA*	ACXXIO_RS09130	LP314_RS09435
GTP diphosphokinase	2.7.6.5	KT	*relA*	ACXXIO_RS09215	LP314_RS09520
L-lactate dehydrogenase	1.1.1.27	C	*ldh*	ACXXIO_RS09825	LP314_RS10140
GTP diphosphokinase	2.7.6.5	S		ACXXIO_RS10135	LP314_RS10475
Protein RecA		L		ACXXIO_RS10620	LP314_RS10970
L-lactate dehydrogenase	1.1.1.27	C	*ldh*	ACXXIO_RS10860	LP314_RS11225
ATP synthase epsilon chain		C		ACXXIO_RS10915	LP314_RS11280
H(+)-transporting two-sector ATPase	3.6.3.14	C	*atpD*	ACXXIO_RS10920	LP314_RS11285
ATP synthase gamma chain		C		ACXXIO_RS10925	LP314_RS11290
H(+)-transporting two-sector ATPase	3.6.3.14	C	*atpA*	ACXXIO_RS10930	LP314_RS11295
ATP synthase subunit delta		C		ACXXIO_RS10935	LP314_RS11300
ATP synthase subunit b		C		ACXXIO_RS10940	LP314_RS11305
ATP synthase subunit		C		ACXXIO_RS10945	LP314_RS11310
ATP synthase subunit		C		ACXXIO_RS10950	LP314_RS11315
Glucose-6-phosphate isomerase	5.3.1.9	G	*pgi*	ACXXIO_RS11440	LP314_RS11855
Lichenan permease IIC component		G		ACXXIO_RS13665	LP314_RS14095

Product	E.C.	COG	Gene	KMU32	KACC 12428^T
Lichenan permease IIC component		G		ACXXIO_RS14135	LP314_RS14570
H(+)/Cl(-) exchange transporter ClcA		P		ACXXIO_RS16715	-
Bile resistance					
Oligopeptide-binding protein OppA		E		ACXXIO_RS00085	LP314_RS00100
Oligopeptide-binding protein OppA		E		ACXXIO_RS00970	-
Glucosamine-6-phosphate deaminase	3.5.99.6	G	*GNPDA*	ACXXIO_RS01090	LP314_RS01200
Dihydrolipoyl dehydrogenase	1.8.1.4	C	*pdhD*	ACXXIO_RS01795	LP314_RS01955
CTP synthase (glutamine hydrolyzing)	6.3.4.2	F	CTPS	ACXXIO_RS02185	LP314_RS02365
UDP-galactopyranose mutase	5.4.99.9	M	*glf*	ACXXIO_RS05400	LP314_RS05620
UDP-galactopyranose mutase	5.4.99.9	M	*glf*	ACXXIO_RS05635	LP314_RS05790
Dihydrolipoyl dehydrogenase	1.8.1.4	C	*pdhD*	ACXXIO_RS05775	LP314_RS05935
Arginine--tRNA ligase	6.1.1.19	J	*argS*	ACXXIO_RS06635	LP314_RS06865
Dihydrolipoyl dehydrogenase	1.8.1.4	C	*pdhD*	ACXXIO_RS09830	LP314_RS10145
Adhesion ability					
Protein-tyrosine-phosphatase	3.1.3.48	T		ACXXIO_RS00355	LP314_RS00485
Exodeoxyribonuclease III	3.1.11.2	L	*xthA*	ACXXIO_RS03840	LP314_RS04025
Chitinase	3.2.1.14	S		ACXXIO_RS04075	LP314_RS04270
Protein-tyrosine-phosphatase	3.1.3.48	GM		ACXXIO_RS05485	LP314_RS05705
Chitinase	3.2.1.14	S		ACXXIO_RS08105	LP314_RS08375
Multidrug resistance protein MdtG		G		ACXXIO_RS09720	LP314_RS10030
Potassium channel		S		ACXXIO_RS11840	LP314_RS12260
Protein-tyrosine-phosphatase	3.1.3.48	T		ACXXIO_RS12455	LP314_RS12880
Protein-tyrosine-phosphatase	3.1.3.48	T		ACXXIO_RS14770	LP314_RS15200
